# Takotsubo cardiomyopathy associated with anesthesia: three case reports

**DOI:** 10.1186/1757-1626-1-227

**Published:** 2008-10-08

**Authors:** F Craig Littlejohn, Omar Syed, Eugene Ornstein, E Sander Connolly, Eric J Heyer

**Affiliations:** 1 Department of Anesthesiology, Columbia University Medical Center, New York, NY, USA; 2 Neurological Surgery, Columbia University Medical Center, New York, NY, USA; 3 Neurology, Columbia University Medical Center, New York, NY, USA

## Abstract

Takotsubo cardiomyopathy is a form of transient, reversible left ventricular dysfunction that can mimic an acute coronary event. However, cardiac catheterization often reveals normal coronary arteries. Patients are often postmenopausal women who experience acute physical or emotional distress. The prognosis for this entity is quite favorable. In this report, we present three cases of Takotsubo cardiomyopathy in patients having procedures involving anesthesia. Each case illustrates a different etiology for the syndrome: Patient, procedure, and anesthetic management.

## Background

Takotsubo cardiomyopathy, also known as the 'transient left ventricular apical ballooning syndrome', is an uncommon phenomenon originally described in the Japanese population in 1991. [1] Sufferers are typically postmenopausal women who experience acute physical or emotional stress and subsequently develop chest pain and dyspnea. [2] The pathophysiological mechanism is likely catecholamine overload. [3]

Ventriculography typically reveals LV apical akinesis/hypokinesis and basal hypercontractility. [4] This gives the characteristic "takotsubo," or octopus trap appearance. Ejection fraction is typically near 30% [3]. Coronary angiography is typically unremarkable, as most patients have no coronary artery disease, or non-obstructive disease [4]. Prognosis is generally favorable, as in-hospital mortality was 1.1%, and recurrence 3.5% in a review of multiple studies. [5] We present three cases of Takotsubo cardiomyopathy occurring at our institution in the past six months in patients having procedures involving anesthesia. Each case illustrates a different etiology for the syndrome: Patient, procedure, and anesthetic management.

## Case presentation

### Case 1

A 71 year-old woman with a history of depression, refractory to medical management, presented with a major depressive episode for electroconvulsive therapy (ECT).

She had an uneventful ECT series four years prior, consisting of approximately 10 sessions. Her past medical history was significant only for hypertension. Her medications included daily quetiapine 300 mg, nortriptyline 25 mg, metoprolol succinate 100 mg, amlodipine 5 mg, and ativan 0.5 mg. She was also taking a four-day course of cefuroxime for a urinary tract infection. She had no allergies, and no family history of heart disease. Her electrocardiogram one day prior to ECT revealed normal sinus rhythm, at 97 beats per minute, with no ST or T abnormalities, or Q waves.

Anesthesia was induced with 50 mg of methohexitol, and 50 mg of succinylcholine. After unilateral ECT was delivered to the right side of the head, a generalized seizure was initiated. The patient remained hemodynamically stable, resumed spontaneous respirations, and emerged rapidly.

Three to four hours following the procedure she complained of chest pain. Her ECG revealed T wave flattening in AVL, and poor precordial R wave progression. A troponin level performed at that time was 3.28 ng/mL. Cardiac catheterization revealed non-obstructive coronary artery disease. Echocardiography demonstrated LV dysfunction with an ejection fraction of 20%, and a large apical aneurysm. A follow up echocardiogram one day later revealed improved LV function, and an EF of 45%. The troponin levels decreased over seven days to 0.18 ng/mL. Based on these results, the consulting cardiologist diagnosed Takotsubo cardiomyopathy. Subsequently, this patient's blood pressure regimen was modified with the addition of an angiotensin converting enzyme inhibitor. Follow-up echocardiography four months later revealed normal ejection fraction. Since that episode, the patient has had nineteen uneventful follow-up ECT treatments. These follow-up ECT treatments were performed with the addition of nicardipine, esmolol, and/or labetolol for blood pressure management, and cardioprotection.

### Case 2

A 34 year-old woman with a history of asthma, hypercholesterolemia, pregnancy associated hypertension, obesity, and migraine headaches presented with a two week episode of post-prandial nausea, vomiting, and right-upper quadrant pain. Cholelithiasis was diagnosed, and the patient was scheduled for elective cholecystectomy. Her medications included daily hydrochlorothiazide 25 mg, rosuvastatin, montelukast sodium 10 mg, twice-daily fluticasone/salmeterol 500/50, and a rarely used albuterol metered-dose inhaler. She recently started celecoxib for her abdominal pain. She had an allergy to simvastatin, which was associated with a rash. She used no alcohol, tobacco, or other drugs. Her family history was significant only for hypertension.

Baseline vital signs included blood pressure 130/74, heart rate 67, respiratory rate 16, and room air oxygen saturation 98%. Exercise tolerance was not limited by chest pain or dyspnea. Her physical exam was significant for obesity, and tenderness to deep palpation of the right upper quadrant, without guarding, or rebound. Preoperative ECG revealed normal sinus rhythm at 64 bpm, normal axis, T inversion in II, III, and AVF, V3-6, with T flattening in V1-2, ST depression of 1 mm in II, II, F, V3-6. A bedside transthoracic echocardiogram was performed in the preoperative area, and was normal. The cardiologist performing the exam felt that the patient was at very low risk for a coronary event, despite the abnormal ECG, and insisted that the patient was in optimal condition for the procedure.

In the operating room, the patient was given intravenous midazolam 1 mg, and fentanyl 50 mcg, and pre-oxygenation. Minutes later she developed pressure-like, non-radiating, substernal chest pain, and dyspnea. She was noted to be diaphoretic, tachypneic, and hypoxic, with an oxygen saturation of 80–90%. Her first non-invasive blood pressure reading was 213/141, with a heart rate in the 140s. Large ST elevations were noted on the 5-lead ECG monitor in leads I, and AVL. Metoprolol 1 mg was given, and the heart rate slowed to the 70s, with an unobtainable blood pressure. Phenylephrine was given, an arterial line was placed, and the initial blood pressure was 90/50. A 12-lead ECG was obtained and revealed new ST elevations in I and AVL, and ST depressions in V4-6.

Surgery was cancelled, and the patient was transferred to the intensive care unit. The initial troponin level was 0.4 ng/mL, and peaked at 9.0 within six hours. The patient received oral aspirin 325 mg, clopidogrel 600 mg, and an intravenous heparin drip. A repeat echocardiogram revealed posterior wall akinesis, inferior wall hypokinesis, and an EF of 35–40%. Cardiac catheterization was performed the following day and revealed normal coronary anatomy. Ventriculography showed findings consistent with the preceding echocardiogram.

After an extensive workup, the patient was diagnosed with Takotsubo cardiomyopathy, with the 0 possibility of vasospastic angina.

A follow-up echocardiogram at one month revealed normal LV size, function, EF, and no wall motion abnormalities. Three months later, the patient had an uneventful laparoscopic cholecystectomy, having started atenolol prior to the procedure.

### Case 3

An 83 year-old woman with bilateral carotid stenosis presented for elective right carotid endarterectomy. Her past medical history included hypertension, hypercholesterolemia, polymyalgia rheumatica, pernicious anemia, and peptic ulcer disease treated with a Billroth 2 gastrectomy. Her preoperative medications included olmesartan, alendronate, rosuvastatin, and aspirin, which had been stopped seven days prior to surgery. Of note, the patient took olmesartan, an angiotensin receptor blocker, the morning of surgery. She had no allergies, no history of smoking, alcohol consumption, or other drug use, and no family history of heart disease.

Her preoperative ECG was significant for low-voltage QRS. Her preoperative blood pressure was 97/58, heart rate 93, respirations 15, and room air oxygen saturation 97%. Her chest was clear to auscultation bilaterally, heart tones were normal, and there was no JVD. No medications were given in the preoperative area.

In the operating room, an IV and radial arterial line were placed, and the patient was sedated with midazolam and fentanyl. Anesthesia induction and intubation were facilitated with lidocaine, etomidate, and vecuronium. Prior to induction, a phenylephrine drip had been started at 25 mcg/min to maintain systolic blood pressure greater than 150 mm Hg, a target requested by the neurosurgical attending. During intubation, blood pressure briefly rose to 220/100 with a heart rate of 100. Subsequently, during dissection of the carotid sheath, blood pressure was noted to be poorly responsive to phenylephrine boluses, however ephedrine was effective. At one point during dissection an episode of bradycardia and hypotension occurred that was treated with 0.2 mg glycopyrrolate, 10 mg ephedrine, and 80 mcg phenylephrine simultaneously as the surgeon's released manual pressure on the carotid sinus. The heart rate quickly normalized, with recovery of blood pressure to previous levels (SBP 140s).

During the following hour, maintaining the target blood pressure became increasingly difficult, and was punctuated by a series of transient precipitous drops, followed by recovery with treatment. Vasopressin was given in two 0.5 unit boluses, with a 2 unit/hr infusion, followed by several boluses of norepinephrine ranging from 8–32 mcg. The phenylephrine infusion started at the beginning of the case was titrated as high as 75 mcg/min, but was safely discontinued upon release of the carotid cross clamp. The procedure was nearing completion by the end of the hour, and pink frothy fluid was noted in the gas sampling line. Breath sounds were coarse, but not crackly. Oxygen saturation fell to 95% at one point, but recovered to 97% following suctioning of the endotracheal tube. The patient was deemed stable for extubation, and was transported to the PACU on a 100% non-rebreather mask.

In the PACU, the patient complained of dull, non-radiating, substernal chest pain. The ECG revealed a low-voltage QRS, but was essentially unchanged from baseline. A chest x-ray revealed pulmonary edema. The troponin level was 5.19 ng/mL. Cardiac catheterization revealed normal coronary anatomy, ventriculography revealed severe anterolateral hypokinesis, and severe apical septal hypokinesis, with a left-ventricular ejection fraction of 25%. Of note, pulmonary capillary wedge pressure was 25 cm H2O. An intra-aortic counterpulsation balloon pump was placed for cardiogenic shock. The patient recovered in coronary care unit (CCU), and was discharged one week later. Follow-up studies cardiac studies were said to have returned to normal.

## Discussion

Takotsubo cardiomyopathy is most commonly associated with acute physical or emotional stress. [2] Our cases are unusual in that they were all associated with different forms of iatrogenic stress related to medical procedures.

First, during the course of ECT treatment, it is common for blood pressure and heart rate to increase because of endogenous catecholamine release. Elevated catecholamine levels are found within one minute and frequently last 10–15 minutes [6,7]. However, there have been only a few case reports of Takotsubo cardiomyopathy associated with ECT [8].

Second, while it is difficult to determine precisely the etiology and sequence of pathological events in our second case, the patient experienced an acutely stressful event, and developed Takotsubo cardiomyopathy. Given this history of migraine headaches, and sudden onset of the episode, we feel it is most likely that this patient experienced severe coronary vasospasm, which may have resulted in ischemia, leading to myocardial dysfunction.

Alternatively, the emotional and physical stress of the coronary vasospasm may have caused a surge in endogenous catecholamines that was directly responsible for inducing Takotsubo cardiomyopathy, as diagnosed by the cardiologist.

Third, the catecholamine excess that led to Takotsubo cardiomyopathy was iatrogenic. To optimize collateral circulation to the brain during carotid cross-clamping, anesthesiologists frequently induce hypertension. Our patient was particularly resistant to the usual doses of phenylephrine, a pure alpha-1 vasoconstrictor. We attributed this to preoperative treatment of her hypertension with an angiotensin receptor blocker (ARB), which has been associated with enhanced and potentially severe hypotension during anesthesia. [6,7] Her blood pressure did respond to ephedrine, an indirect inotrope-vasoconstrictor. She received a total of 50 mg ephedrine over the first hour of surgery. The phenylephrine, ephedrine, norepinephrine and vasopressin likely all contributed to the patient developing Takotsubo cardiomyopathy, and ultimately heart failure. The most effective treatment for anesthesia-induced ARB/ACE-I hypotension is terlipressin. [8,9] In this case, vasopressin was given in two small boluses, and an infusion was started. At the same time, several boluses of norepinephrine were given. These treatments were minimally effective, because she was likely already in heart failure.

Takotsubo cardiomyopathy is an increasingly reported phenomenon that may occur not only in settings of acute physical or emotional stress, but also iatrogenic stress related to common medical procedures. Physicians participating in these procedures would be well advised to consider the risk factors inherent in the patient, procedure, and anesthetic management for developing this syndrome, in order to better anticipate and potentially prevent this outcome.

## Consent

A telephone informed consent including witness in accordance with the author's local institutional guidelines was obtained from each patient for publication of this case report. A copy of this consent is available for review by the Editor-in-Chief of this journal.

## Competing interests

The authors declare that they have no competing interests.

## Authors' contributions

FCL and ONS prepared the manuscript. EO and EJH were the anesthesiologists caring for these patients. ESC was the neurosurgeon involved for patient three. Collectively, EO EJH, and ESC analyzed the data and provided critical assessment for the final manuscript. All authors read and approved the final manuscript.
